# Complexity of Social Networks

**DOI:** 10.3390/e28091008

**Published:** 2026-09-09

**Authors:** Zi-Ke Zhang, Junming Huang, Xiaoke Xu, Quanhui Liu

**Affiliations:** 1 College of Media and International Culture, Zhejiang University, Hangzhou 310058, China; 2 Paul and Marcia Wythes Center on Contemporary China, Princeton University, Princeton, NJ 08544, USA; junminghuang@princeton.edu; 3 Center for Computational Communication Research (Zhuhai), School of Journalism and Communication, Beijing Normal University, Beijing 100875, China; xuxiaoke@foxmail.com; 4 College of Computer Science, Sichuan University, Chengdu 610065, China; quanhuiliu@scu.edu.cn

Research on social networks now sits at a productive intersection between network science and computational social science [1]. Digital traces have made relational processes observable at a scale and temporal resolution that were difficult to reach with earlier data, while recent developments in higher-order and multilayer networks have widened the range of interactions that can be represented [2,3,4,5]. Advances in computational text analysis have further strengthened the connection between relational structure and the meanings carried by communication [6]. Studies of event diffusion and temporal intervention illustrate how current research increasingly follows networks as evolving processes [7,8]. These developments have created substantial analytical opportunities. They have also made questions of measurement more consequential.

The 17 contributions in this Special Issue approach this problem from markedly different domains, including online communication, collective behavior, public health, financial markets, trade, and transportation. Their deep connection lies in a shared concern with relational dependence and with the conditions under which observed network patterns acquire substantive meaning. Across the collection, networks serve as more than fixed settings in which social processes unfold. They provide representations whose interpretation depends on how relations are defined, how interactions evolve, and how observations are connected to the mechanisms under study. The contributions suggest that one of the central challenges in contemporary network research is to understand the conditions under which such representations remain informative as the systems behind them change.

One consequence concerns measurement. Network analysis often begins from an observed graph, although that graph already embodies choices about sampling, estimation, aggregation, and representation. Tan et al. (Contribution 1) use evidence theory to integrate multiple indicators in influential-node identification, while Wen et al. (Contribution 2) examine biases in ego-centric network estimation under degree and attribute dependence. Hashimoto et al. extend the problem into temporal settings by examining the robustness of community membership under structural perturbations in Contribution 3. Although these studies address different problems, they all make clear that uncertainty enters network analysis before structural quantities are ever computed. Structural findings therefore become more informative when the assumptions that produced the underlying representation are made explicit and their consequences can be assessed. This issue is likely to become increasingly important as network data grow in scale while also becoming more heterogeneous in origin and quality.

The relationship between structure and dynamics raises a related challenge. Luo et al. (Contribution 4) use empirically calibrated branching processes to characterize information cascades, while Rodrigues et al. (Contribution 5) show how inertia and directionality alter the phase behavior of a majority-vote model. Contribution 6, Contribution 7, and Contribution 8 connect relational patterns with attitudinal similarity, emotional change, and moral contagion. Huang et al. examine the longitudinal co-evolution of narratives across a hybrid information environment in Contribution 9, and Peláez et al. (Contribution 10) develop an entropic perspective on divergence among public information sources. These studies illustrate why relational structure and the processes associated with it often need to be analyzed jointly. Their results also call for caution in interpretation. Observed associations among network position and behavior should not automatically be interpreted as evidence of causal feedback, and the reproduction of aggregate network patterns does not necessarily identify a unique underlying mechanism.

Computational measurement creates a similar opportunity and limitation. Contribution 11 and Contribution 12 develop learning frameworks for detecting forms of harmful or implicit language, while Hu et al. (Contribution 13) combine multidimensional analysis with entropy-based measures to characterize systematic linguistic variation. Such approaches expand the scale and sensitivity of computational measurement in communication research. Predictive performance represents a different scientific achievement from explanation. A model may identify a linguistic category with high accuracy while leaving unresolved why the observed pattern emerged, whether the same relationship will persist as the communication environment changes, and how strongly the result depends on the data from which the measurement was learned. The growing power of computational measurement therefore increases the importance of validating whether the resulting quantities remain interpretable across populations, platforms, and periods of observation.

The same concern becomes harder to ignore when similar network methods are applied to systems with very different meanings of connection. Gong and Wang examine tail-risk spillovers between cryptocurrency and energy markets in Contribution 14. Wang et al. (Contribution 15) model coupled vaccination behavior and epidemic spreading using multilayer higher-order networks. Zhou et al. (Contribution 16) study vulnerability and cascades in the global railway-vans trade network, while Ren et al. (Contribution 17) examine disruption propagation in a geopolitical multiplex representation of container shipping. These systems differ substantially in the processes that generate their edges and in the meaning of a connection. These studies illustrate how relational dependence can shape the propagation and distribution of system-level effects. At the same time, this breadth creates a conceptual obligation. As network methods travel across domains, researchers need to make clear what the network representation contributes to explanation and which conclusions arise from domain-specific assumptions. A network can provide a common analytical language, while the mechanisms attached to its nodes, edges, and layers remain specific to the system being studied.

Network construction consequently deserves to be treated as part of inference itself. Choices concerning edge definitions, temporal windows, thresholds, or layers determine which relational patterns can become visible and can sometimes alter the macroscopic structure inferred from the same underlying activity. The contributions in this Special Issue address different parts of this problem through uncertainty modeling, temporal analysis, higher-order interactions, and multiplex representations. A useful next step is to make representational robustness more systematic. Findings that persist across reasonable alternative constructions provide stronger evidence of structural regularity, whereas findings that change substantially with representation may reveal where substantive conclusions depend on analytical choices. Making this dependence visible is especially important when several plausible networks can be constructed from the same digital traces.

Several contributions also point toward questions of intervention. Influential-node identification, harmful-content detection, diffusion modeling, and vulnerability analysis all have evident practical applications. Once such models are used to guide action, however, the system being modeled may respond to the intervention itself. Targeting particular actors can redistribute attention, while interventions in communication or infrastructure networks can change the flows from which later network measurements are constructed. Evaluation in these settings therefore needs to consider adaptation after intervention rather than only immediate predictive accuracy or local optimization. This problem is likely to become more important in systems where individuals, organizations, and platforms respond strategically to changes in their environment.

Several developments beyond this collection are likely to shape the next stage of the field. Higher-order models are expanding the range of collective interactions that network analysis can represent, while empirical identification of such interactions remains challenging. Narrative diffusion offers another direction because meaning can change as information moves through a network [9]. Artificial intelligence adds a further complication. Large language models are entering computational social science as tools for classification, simulation, and data generation [10], and platform algorithms already influence the traces from which networks are reconstructed [11]. As generative and recommendation systems become more directly involved in communication, future network data will increasingly mix human activity with algorithmic selection and machine-generated content. Comparisons across platforms and periods will therefore require careful documentation of the data-generating environment.

Progress will also depend on stronger links between network description and social explanation. Work on digital trace quality and methodological standards shows that computationally precise measures can still carry substantial uncertainty about validity and interpretation [12,13,14]. For social-network research, causal claims should remain proportionate to the research design, and competing explanations deserve consideration even when structural patterns are stable. Longitudinal evidence, natural experiments, multimethod validation, and sensitivity analyses across alternative network constructions can help narrow this gap. The contributions in this Special Issue show why these issues matter across communication, public health, markets, and infrastructure. Their value lies in placing different forms of relational complexity within a shared analytical conversation. We hope that the collection encourages further work in which network models remain mathematically informative and socially interpretable as the systems under study continue to change.
